# Flaxseed Can Reduce Hypoxia-Induced Damages in Rat Testes 

**DOI:** 10.22074/ijfs.2018.5298

**Published:** 2018-06-20

**Authors:** Mahnaz Poorhassan, Fatemeh Navae, Simin Mahakizadeh, Mahshid Bazrafkan, Banafshe Nikmehr, Farid Abolhassani, Sahar Ijaz, Nazila Yamini, Nasrin Dashti, Kobra Mehrannia, Gholamreza Hassanzadeh, Mohammad Akbari

**Affiliations:** 1 Department of Anatomy, School of Medicine, Tehran University of Medical Sciences, Tehran, Iran; 2 Department of Clinical Laboratory Sciences, School of Allied Medical Sciences, Tehran University of Medical Sciences, Tehran, Iran

**Keywords:** Flaxseed, Hypoxia, Rat, Sperm, Testis

## Abstract

**Background:**

Hypoxia causes detrimental effects on the structure and function of tissues through increased production of reactive oxygen species that are generated during the re-oxygenation phase of intermittent and continuous hypobaric hypoxia. This study was carried out to evaluate the effects of flaxseed (Fx) in reducing the incidence of hypoxia in rat testes.

**Materials and Methods:**

In this experimental study, 24 adult Wistar rats were randomly divided into four groups: i. Control group (Co) that received normal levels of oxygen and food, ii. Sham group (Sh) that were placed in hypoxia chamber but received normal oxygen and food, iii. Hypoxia induction group (Hx) that were placed in hypoxia chamber and treated with normal food, iv. Hypoxia induction group (Hx+Fx) that were placed in hypoxia chamber and treated with 10% flaxseed food. Both the Hx and Hx+Fx groups were kept in a hypoxic chamber for 30 days; during this period rats were exposed to reduced pressure (oxygen 8% and nitrogen 92%) for 4 hours/day. Then, all animal were sacrificed and their testes were removed. Malondialdehyde (MDA) and total antioxidant capacity (TAC) levels were evaluated in the testis tissue. Tubular damages were examined using histological studies. Blood samples and sperm were collected to assess IL-18 level and measure sperms parameters, respectively. All data were analyzed using SPPSS-22 software. One way-ANOVA or Kruskal-Wallis tests were performed for statistical analysis.

**Results:**

A significant difference was recorded in the testicular mass/body weight ratio in Hx and Hx+Fx groups in comparison to the control (P=0.003 and 0.027, respectively) and Sh (P=0.001 and 0.009, respectively) groups. The sperm count and motility in Hx+Fx group were significantly different from those of the Hx group (P=0.0001 and 0.028, respectively) .Also sperm viability (P=0.0001) and abnormality (P=0.0001) in Hx+Fx group were significantly different from Hx group.

**Conclusion:**

This study therefore suggests that the oral administration of flaxseed can be useful for prevention from the detrimental effects of hypoxia on rat testes structure and sperm parameters.

## Introduction

Hypoxic conditions can be found in many situations such as high altitude, diving, and chronic obstructive pulmonary disease (COPD). Globally, COPD is considered as a leading cause of death and disability (1). Hypoxic conditions result in lower levels of circulating oxygen (2) and 4-week exposure to hypoxia produces systemic hypoxia in rats as manifested by pulmonary hypertension, and increased right ventricular systolic pressure (3). These hypoxic signs present special challenges to homeostasis because of their effects on sympathetic outflow and vascular smooth muscle.

It is generally accepted that chronic systemic hypoxia, whether due to high altitude or imposed experimentally by a hypoxic or hypobaric chamber, induces physiological adaptations that help to compensate the impaired O_2_ transport to tissues. Enhancing red blood cell production (e.g.by administration of erythropoietin (Epo) has been shown to modulate the ventilatory response to reduced oxygen supply and critically help the organism to cope with increased oxygen demand (4). Exposure to hypoxia has been associated with an increase in the production of reactive oxygen species (ROS) that are generated during the re-oxygenation phase of intermittent and continuous hypobaric hypoxia and contribute to physiological responses (5) such as pulmonary hypertension and vasoconstriction as well as neomuscularization and thickening of the media and adventitia of pulmonary arterioles.

Weight loss due to exposure to chronic hypoxia may reflect multiple changes in cardiovascular function, hormone production, energy metabolism, and other aspects of cellular and systemic physiology (4). ROS may cause cell membrane damage, and prevent the maintenance of ionic gradient which can lead to detrimental effects on structure and function of tissues (6, 7), impairment in ATP production and tissue inflammation. Oxidative stress (OS) refers to an imbalance between generation of ROS and the ability of endogenous antioxidant systems to scavenge ROS, where ROS overwhelms antioxidant capacity (5, 8).

Furthermore hypoxic condition increases the levels of inflammatory cytokine such as IL-1ß, IL-18 and tumor necrosis factor-alpha (TNF-α) (9). Also, hypoxia increases levels of lipid peroxidation-while reduces glutathione reductase activity and number of epididymal sperm (10). Evident changes observed following hypoxia-induced lipid peroxidation have been reported (11). These changes are partially attenuated by supplementation of antioxidants such as melatonin and ascorbate but there is no report about the effect of flaxseed on male reproductive system affected by hypoxia. The major components of flaxseed are the essential n-3 fatty acid, a-linolenic acid (ALA), lignans such as secoisolariciresinol diglucoside (SDG) and carbohydrates such as mucilages containing arabinoxylans. ALA is orally bioavailable and may be stored or converted into longer chain n-3 fatty acids such as eicosapentaenoic acid (EPA) and docosahexaenoic acid (DHA) and other bioactive lipid metabolites (12). SDG is metabolized to the mammalian lignans, enterodiol and enterolactone, in the intestine (13); recent research has demonstrated the ability of lignans to scavenge hydroxyl radicals suggesting a potent antioxidant activity for lignans. lignans are biologically active phytochemicals with anticancer and antioxidant potential (14). Docosahexaenoic acid has been shown to increase sperm motility in men (15). 

Improvement of vascular endothelial cell function, enhancement of vascular reactivity and compliance, modulation of lipid metabolism and reduction of inflammatory cytokine production have been noted as the underlying mechanisms through which poly unsaturated fatty acid (PUFA) exert their beneficial effects (16) . In mammalian sperm, lipids especially n-3 fatty acids are dominantly present. Previous studies have shown that n-3 fatty acids are also present in human sperm (15). Their protective mechanisms include induction of anti-inflammatory transcriptional pathways , reducing the intracellular Ca^2+^ levels, suppression of vascular proliferation, and improvement of cell membrane integrity (17). Little information is available regarding the effect of dietary flaxseed supplementation on male rats’ reproductive system following exposure to hypoxia. The objective of the present study was to investigate the effect of flaxseed supplementation on testes structure and sperm parameters of hypoxic rats.

## Materials and Methods

In this experimental study, 24 male Wistar albino rats (270-300 g, 12-weeks-old) were purchased from Pharmacy Faculty of Tehran University of Medical Sciences, Tehran, Iran. Animals were allowed to have access to food and water. Also, they were kept under 12- hour periods of light and darkness at 23 ± 2°C. All procedures were carried out in accordance with the guidelines of the Iranian Council for use and care of animals and approved by Ethics Committee of Tehran University of Medical Sciences.

### Experimental design

The rats were randomly divided into 4 groups: control (Co), sham (Sh), hypoxia (Hx) and hypoxia+flaxseed (Hx+Fx). Hypoxic rats were kept in a hypoxic chamber with a reduced pressure (oxygen 8% and nitrogen 92% for 4 hours/day for 30 days). The reason for using 8% oxygen is that the rats are capable to survive at this level of hypoxia which allows us to measure the patho-physiologic variables in them (18).

Control group (Co) was kept under normoxia and had free access to standard food and water. Sham group (Sh) was maintained in a hypoxia chamber (but not under hypoxia) receiving normal oxygen and food. Hypoxia group (Hx) was exposed to hypoxia 4 hours/day and fed with normal food. Hx+Fx group: 10% Fx was added to the normal food of Hx+Fx group after the first hypoxic exposure. 

### Testis index

At the end of the experimental period, each rat was weighed and sacrified. Then, the right testis was removed and weighed. The testicular mass relative to body weight was determined on day 42 using the following equation: (testicular/body weight ratio)*100=(%).

### Detection of IL-18 levels

At the end of each experiment, blood samples were collected from the left ventricle. Blood was centrifuged at 1000 g for 15 minutes and serum was separated for biochemical analysis. IL-18 levels in serum samples were quantified by an ELISA kit (zell Bio-GmbH, Germany) according to the manufacturer’s instructions.

### Histological procedure

At the end of the experiment, rats were weighed and sacrificed and their right testis was removed. The right testicular (internal spermatic) vein drained directly into the right common iliac vein in 77.4%, and into the inferior vena cava in 22.6% of the animals. The left testicular vein drained into the left common iliac vein in all animals, but in 90.3% of rats there was also an accessory branch of the testicular vein draining into the left renal vein (19). Testes were placed in Bouin’s solution for 24 hours at room temperature. Later, they were processed, sectioned and stained with H&E technique. On slices with 5- µm thickness, the morphometric assessment of seminiferous tubules was performed. The tubular diameters and germinal epithelial thickness of seminiferous tubules that were sectioned transversely were evaluated using light microscopy (20). In this way, the slides were studied at ×100 magnifications, and in different fields of testis tissue, 20 tubules from each specimen were studied. The analyses were carried out on images were taken using LABOMED digital camera (LABOMED, USA). Then, the images were processed by the image analysis system software of Image J (ImageJ U. S. National Institutes of Health, Bethesda, Maryland, USA). Finally, the scale bar was added to the images (21).

### Sperm sampling

The caudal epididymis was used for sperm analysis. Briefly, epididiymal sperms were collected by slicing the caudal epididymis in 1 ml of Minimum Essential Medium-a (MEM-a) medium (P/N 22561-021, Gibco, CA, USA) after that 9-ml medium was added and samples were incubated for 10 minutes to allow the sperms to swim into the medium. The epididymis was then processed for further analysis.

### Sperm count

To enumerate the spermatozoa, the heads of spermatozoa were counted. For sperm counting, a hemocytometer device was used. Here, 50 µl of the suspension was mixed with an equal volume of 2% formalin. Then, 10 µl of this diluted suspension was transferred to a Neubauer chamber. The sperms were counted under light microscopy at ×400 (22).

### Sperm morphology

A part of sperm sample was used for preparing smears to evaluate the sperm morphological abnormalities. For this purpose, 10 µL of suspension was spread onto a glass slide and allowed to air-dry at room temperature to prepare a smear. The smears were then stained with Diff- Quik stain and 200 sperms were then examined under light microscopy at ×400 (22).

### Sperm viability assay

In order to study the sperm viability, 10 µl of sperm suspension was mixed with 2 µl Eosin-y 0.05%. Slides were prepared and incubated for two minutes at room temperature before evaluation at ×400 magnifications using light microscopy. Two hundred sperms were counted for each sample. Dead sperms appeared pink and live sperms were not stained (22).

### Sperm motility

One to two drops of the sperm suspension were placed on a glass slide and motile sperms were counted immediately using light microscopy (22).

### Tissue preparation for enzyme assay

Rat testes were rapidly removed and manually homogenized in cold phosphate buffer (pH=7.4) and debris was removed by centrifugation at 3500 g for 10 minutes. Then, 50 mg of supernatant was homogenized in 10 volumes of KH2PO4 (100 mmol) buffer and was centrifuged at 12,000 g for 30 minutes at 4ºC. The supernatant was collected and used for enzymes and MDA levels studies (23).

### Measurement of total anti-oxidant capacity and lipid peroxidation

Total antioxidant capacity was measured based on the absorbance of the 2,2'-azinobis-3-ethylbenzothiazoline- 6-sulfonic acid (ABTS+) radical cation. The pre-formed radical monocation ± of 2,2'-azinobis-(3-ethylbenzothiazoline- 6-sulfonic acid) (ABTS•+) is generated by oxidation of ABTS with potassium persulfate and is reduced in the presence of such hydrogen-donating antioxidants. The influences of both the concentration of a given antioxidant and duration of reaction on the inhibition of the radical cation absorption are taken into account when determining the antioxidant activity (24). A common method for measuring MDA, referred to as the thiobarbituric acid- reactive-substances (TBARS) assay, is based on its reaction with Thiobarbituric acid (TBA) followed by reading the absorbance at 532 nm. Thiobarbituric acid substance assay is a method to quantify malondialdehyde concentration by spectrophotometry (25).

### Statistical analyses

Data were statistically analyzed using SPSS-22 (IBM crop., Armonk, NY, USA) software. All data were expressed as mean ± standard errors of mean (SEM), median and interquartile range (IQR). At first, the normality of variables was checked using the Kolmogorov-Smirnov test. Then, for analyzing the differences among four groups of study, one way-ANOVA test and Tukey-post hoc test were chosen if the distribution of data were normal (for sperm parameters, testicular/ body weight ratio, diameter of seminiferous tubules, MDA level and TAC). Otherwise, nonparametric test of Kruskal-Wallis was carried out (for thickness of the germinal epithelium). The statistical significance level was set at 0.05.

## Results

### Model confirmation

**Fig.1:**
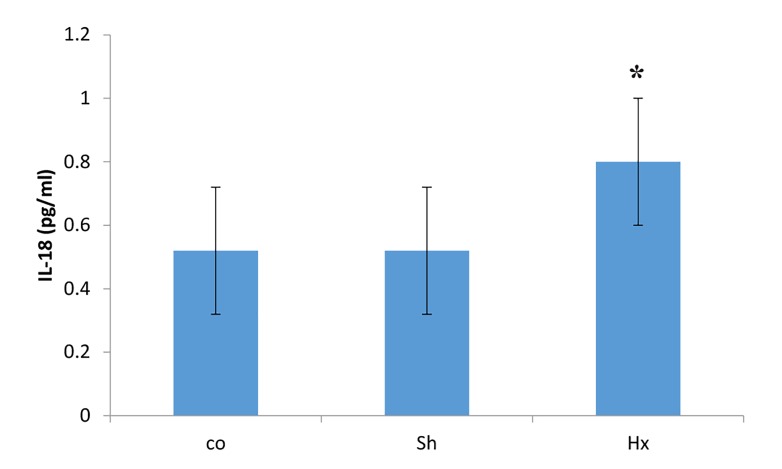
Effects of hypoxia on serum levels of IL-18 (pg/ml) in rats following hypoxia. *; P<0.05 compared to control and sham groups, Co; Normal group that received normal oxygen levels and normal food, Sh; Sham group maintained in hypoxia chamber with normal oxygen levels and food, and Hx; Animals were exposed to hypoxia and received normal food.

Using one way-ANOVA test, serum levels of IL-18 were compared to confirm state of hypoxia. Tukey post hoc test showed a significant difference in serum levels of IL-18 in rat exposed to 30-days hypoxia (0.08 ± 0.05 pg/ ml) compared to control (0.51 ± 0.08 pg/ml, P=0.0001) and Sham (0.52 ± 0.08 pg/ml, P=0.0001) groups (Fig .1).

### Effects of flaxseed on the body weight and testicular mass/body weight ratio in rats with hypoxia

**Fig.2:**
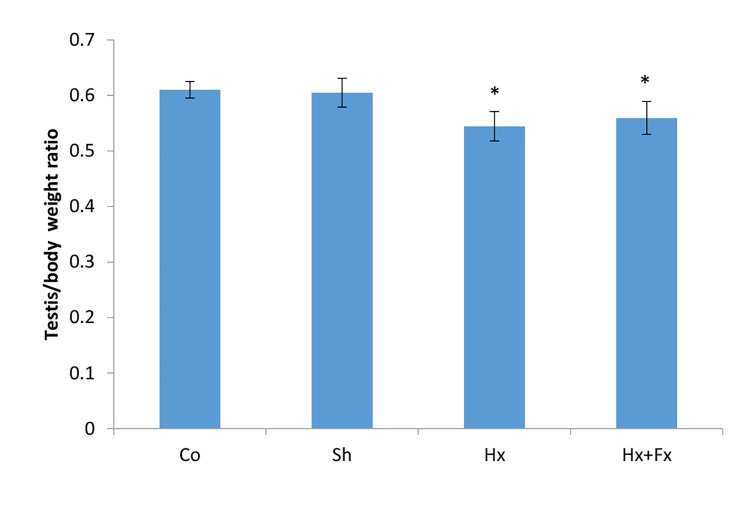
Effects of oral flaxseed on testicular mass/body weight ratio in rats following hypoxia. *; P<0.05 compared to control and sham groups, Co; Normal group that received normal oxygen levels and normal food, Sh; Sham group maintained in a hypoxia chamber with normal oxygen levels and food, Hx; Animals were exposed to hypoxia and received normal food, and Hx+Fx; Animals were exposed to hypoxia and treated by normal food supplemented with 10% Fx.

The effect of oral Fx on the testicular/body weight ratio was evaluated in rats after hypoxia. According to the ANOVA test, the testicular mass/body weight were significantly different in the studied groups (P=0.0001, Fig .2). A significant difference was observed in the testicular mass/body weight of Hx (0.54 ± 0.01%) and Hx+Fx (0.56 ± 0.1%) groups compared to control (0.6 ± 0.1%, P=0.003 and P=0.027, respectively) and sham (0.61 ± 0.1%, P=0.001 and P=0.009, respectively) groups (Fig .2). 

### Effects of flaxseed on sperm parameters in rats exposed to hypoxia 

**Fig.3:**
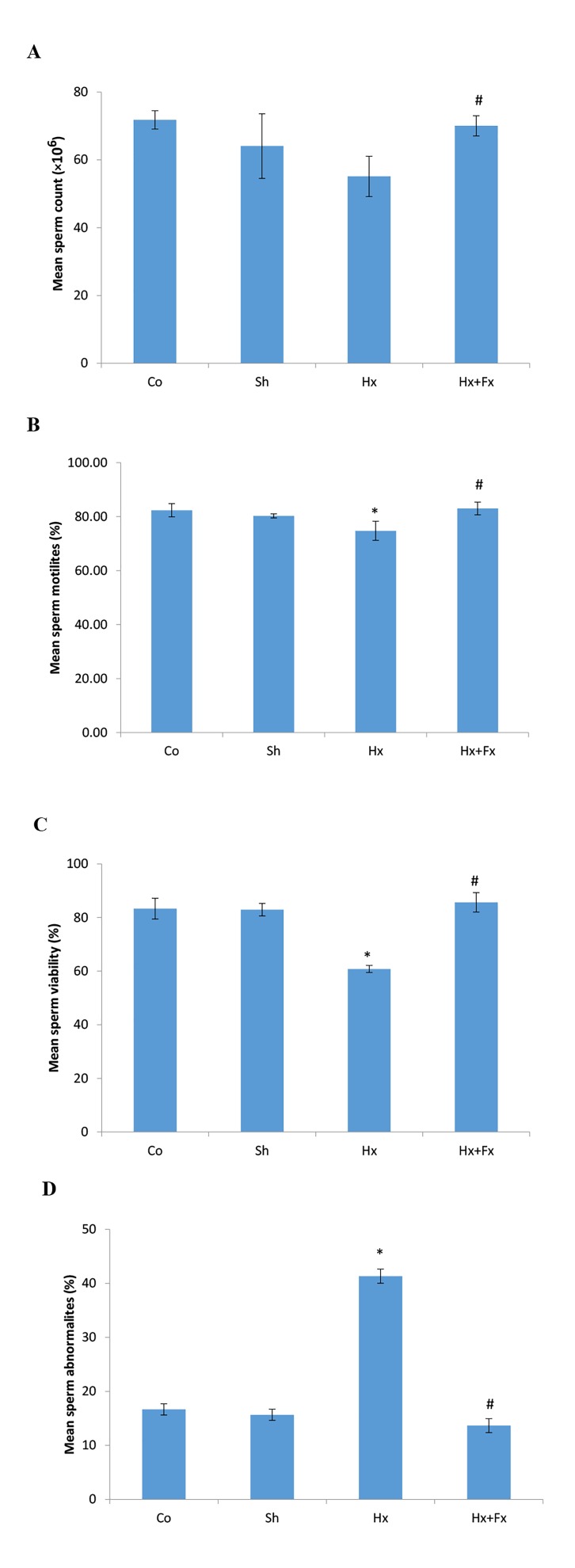
Effects of oral flaxseed on sperm parameters of rats following hypoxia. A. Sperm count, B. Sperm motility, C. Sperm viability, and D. Sperm abnormality. *; P<0.05 compared to control and sham groups, #; P<0.05 compared to HX group, Co; Normal group that received normal oxygen levels and normal food, Sh; Sham group maintained in hypoxia chamber with normal oxygen levels and food, Hx; Animals were exposed to hypoxia and received normal food, and Hx+Fx; Animals were exposed to hypoxia and received normal food supplemented with 10% Fx food.

The effects of oral Fx on sperm parameters were evaluated in rats after hypoxia. The mean sperm count was significantly different in the studied groups (P=0.0001, Fig .3). A significant difference (P=0.0001) was observed in the sperm count between Hx+Fx group (73.02 ± 1.93) and the Hx group (55.12 ± 3.84) (control=71.78 ± 0.22 and Sham=64.06 ± 6.14) (Fig .3). Moreover, the mean sperm motility was significantly different among the studied groups (P=0.025, Fig .3). A significant difference was found in sperm motility between Hx group (74.76 ± 2.27%) and the control (82.35 ± 1.59%, P=0.032) and sham (80.47 ± 0.67%, P=0.041) groups (P<0.05, Fig .3). Also, a significant difference was observed in the sperm motility between Hx+Fx group (83.04 ± 1.52%) and the Hx group (P=0.028, Fig .3). Based on ANOVA test, a significant difference was found in sperm viability between Hx group (60.8 ± 0.85%) and control (83.31 ± 2.5%, P=0.0001) and sham (82.92 ± 1.5%, P=0.0001) groups (Fig .3) and a significant difference was observed in the sperm viability between Hx+Fx group (85.67 ± 1.33%) and the Hx group (P=0.0001, Fig .3). The mean sperm abnormality was significantly different among the studied groups (P=0.0001, Fig .3). A significant difference was seen in sperm abnormality between Hx group (41 ± 1%) and control (17 ± 1.1%, P=0.0001) and sham (16 ± 1.3%, P=0.0001) groups (Fig .3) and a significant difference was observed in the sperm abnormality between Hx+Fx group (14 ± 1.2%) and Hx group (P=0.0001, Fig .3).

### Effects of flaxseed on diameter of seminiferous tubules and thickness of the germinal epithelium in rats exposed to hypoxia

**Fig.4:**
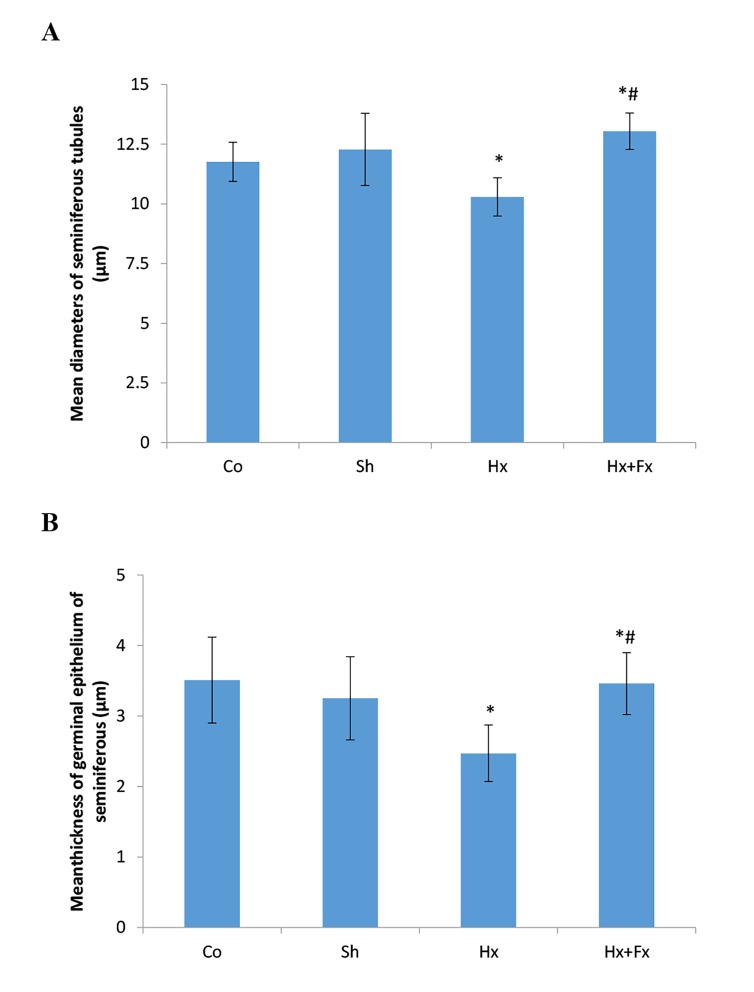
Effects of flaxseed on diameter of seminiferous tubules and thickness of the germinal epithelium in rats exposed to hypoxia. Comparing A. The diameter of seminiferous tubules and B. Thickness of the germinal epithelium in different groups. *; P<0.05 compared to Control and Sham groups, #; P<0.05 compared to Hx group, Co; Normal group that received normal oxygen levels and normal food, Sh; Sham group maintained in hypoxia chamber with normal oxygen levels and food, Hx; Animals were exposed to hypoxia and received normal food, and Hx+Fx; Animals were exposed to hypoxia and received normal food supplementated with 10% Fx food.

The effects of oral Fx on the diameter of seminiferous tubules and thickness of the germinal epithelium were evaluated after hypoxia in rats. According to ANOVA test, the mean diameter of seminiferous tubules was significantly different in the studied groups compared to control and sham (P=0.0001, Fig .4). A significant difference was found in the diameter of seminiferous tubules of Hx group (10.58 ± 0.34 µm) in comparison to the control (11.77 ± 0.22 µm, P=0.031) and sham (12.28 ± 0.4 µm, P=0.001) groups (Fig .4) and a significant difference was observed in diameter of seminiferous tubules of Hx+Fx group (13.04 ± 0.2 µm) as compared to the Control (P=0.022), sham (P=0.048) and Hx (P=0.0001) groups (Fig .4). The thickness of the germinal epithelium was significantly different among the studied groups (P=0.008, Fig .4). A significant difference was observed in the thickness of the germinal epithelium of Hx+Fx [3.5 (IQR: 3.13-3.83) µm] group as compared to the Hx [2.28 (IQR:2-2.56) µm, P=0.005] group (Fig .4). 

### The effects of oral flaxseed on MDA and TAC concentrations were evaluated after hypoxia in rats exposed to after hypoxia

**Fig.5:**
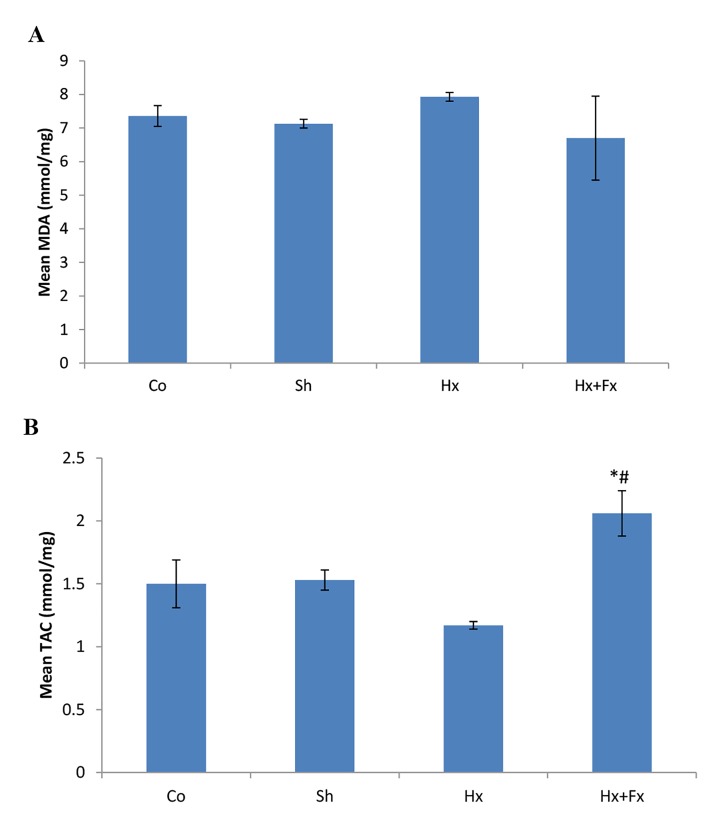
Effects of oral flaxseed on MDA and TAC concentrations in rats exposed to hypoxia. A. MDA and B. TAC concentrations of rats following hypoxia. MDA; Malondialdehyde, TAC; Total antioxidant capacity, *; P<0.05 compared to Control and Sham groups, #; P<0.05 compared to Hx group, Co; Normal group normal oxygen and normal food, Sh; Sham group maintained in hypoxia chamber with normal oxygen and food, Hx; Animals were exposed to hypoxia and received normal food, and Hx+Fx; Animals were exposed to hypoxia and received normal food supplementated with 10% Fx food.

No significant difference was observed in the mean MDA among studied groups (control=7.78 ± 0.11 nmol/mg and sham=7.13 ± 0.09 nmol/mg, Hx=8.57 ± 0.28 nmol/mg and Hx+Fx=6.7 ± 0.81 nmol/mg) (P=0.075, Fig .5). The mean TAC was significantly different among the studied groups (P=0.01, Fig .5). A significant difference was observed in TAC of Hx+Fx (2.07 ± 0.12 nmol/mg) group compared to control (1.51 ± 0.13 nmol/mg, P=0.011), sham (1.53 ± 0.06 nmol/mg, P=0.014) and Hx (1.18 ± 0.02 nmol/mg, P=0.001) groups (Fig .5).

## Discussion

Hypoxia is a condition can result in overproduction of ROS which along with a decrease in the level of antioxidants, may give rise to oxidative stress. Oxidative stress as an imbalance between generation of ROS and ability of endogenous antioxidant systems to scavenge ROS has adverse influence on testes structure and sperm parameters.

In this study, we found that hypoxia leads to reduction in the germinal epithelial thickness and some changes in the serum, testes and sperm parameters in rats also hypoxia results in excessive formation of ROS. We also observed that hypoxia increases interstitial space of the testes, which extends the oxygen diffusion distance and impairs oxygen delivery to germ cells. It makes germ cells more susceptible to damage, which was confirmed by our observation concerning degeneration of germ cells in hypoxic rats. A similar outcome was reported by other researchers (26). In the present study, we observed that flaxseed improves testicular structure as reflected by increased diameter of seminiferous tubules of Hx+Fx group as compared to the Hx group and increased thickness of the germinal epithelium of Hx+Fx group as compared to the Hx group.

Spermatogenesis is vulnerable to hypoxia because spermatogenesis has a high proliferation rate, damanding notable oxygen levels in the testes and it has been reported that breathing 10% O_2_/90% N_2_ results in a 24% decrease in testicular blood flow, but a 23% increase in cerebral blood flow. These characteristics may attribute to the morphological changes of spermatogenesis induced by hypoxia. Besides, a significant decrease in testicular mass followed by adverse effects on reproductive hormones such as testosterone was observed (27). In this study the sperm count, motility and viability significantly decreased in Hx, but increased in Hx+Fx group which might indicate that hypoxia affects sperm sperm differentiation process. We found that flaxseed can improve sperm parameters following exposure to hypoxia. 

In our study there was significant reduction in body weight of Hx+Fx group in comparison to the control and sham groups. Researchers have observed that doses of 5 and 10 g of flaxseed fibers result in prolonged decrease in the levels of ghrelin a hunger-signaling gut peptide (29). 

Dissimilar to many other cell types, sperm lipid membranes contain an exceptionally high percentage of polyunsaturated fatty acids (PUFAs) that provide the fluidity to the membrane contraction events associated with fertilization. However, PUFAs are readily oxidized and produce malondialdehyde.

We reported that lipid peroxidation assessed by MDA levels in all groups exposed to hypoxia was increase but the differences among different groups were not significant. The hypoxia-induced changes in lipid metabolism were mediated via hepatic stearoyl coenzyme A desaturase (25, 30). Lipid peroxidation in mice exposed to sever hypoxia is different from those exposed to moderate hypoxia and the degree of lipid peroxidation rate depend on hypoxia intensity (30). Therefore, probably due to this reason, our result is different from other those of reports. These adverse effects of hypoxia have also been reported to decreased the supplementation of antioxidants such as melatonin and ascorbate (31).

This study shows an increase in serum inflammatory markers (i.e.IL-18) only in group who expose to hypoxia and higher levels of lipid peroxidation and reduces antioxidant activity. In addition, we found flaxseed could effectively counteract peroxidation damage, mediated by the attenuation of systemic and tissue oxidative stress induced by Hypoxia. This is reflected by an increase in TAC values in Hx+Fx group as compared to the Hx group. This is in agreements with previous studies (26).

A high rate of death was observed among animals during the last time of hypoxia procedure.

To confirm the results of this study, we suggest to evaluate the testicular tissue superoxide dismutase (SOD), catalase (CAT), glutathione peroxidase (GPx), glutathione reductase (GRD), and glutathion-S-transferase (GST) activities to confirm the obtained findings.

## Conclusion

The conclusion the present study revealed that flaxseed as an antioxidant drug can reduce hypoxia-induced damages in the testes.
